# Micronutrient composition and acceptability of *Moringa oleifera* leaf‐fortified dishes by children in Ada‐East district, Ghana

**DOI:** 10.1002/fsn3.395

**Published:** 2016-07-05

**Authors:** Mary Glover‐Amengor, Richmond Aryeetey, Edwin Afari, Alexander Nyarko

**Affiliations:** ^1^ Council for Scientific and Industrial Research‐Food Research InstituteAccraGhana; ^2^ School of Public HealthCollege of Health SciencesUniversity of GhanaLegonGhana; ^3^ School of PharmacyCollege of Health SciencesUniversity of GhanaLegonGhana

**Keywords:** Food fortification, green leafy vegetables, local dishes, *M. oleifera*, micronutrients, vitamin A, *β*‐carotene

## Abstract

*Moringa oleifera (M. oleifera)*, a green leafy vegetable, is a good source of minerals and vitamins which could be consumed as part of diet to improve human health and well‐being. Drying *M. oleifera* leaves could make it readily available for use as a food fortificant. The objectives of the study were to determine micromineral and *β* ‐carotene content of dried *M. oleifera* leaves, and leaf‐incorporated local dishes, and also assess school children's (4‐12 yr) acceptability of dried *M. oleifera* leaf‐incorporated local dishes and feasibility of introducing dried leaves into a school lunch menu. *M. oleifera* leaves were solar dried, milled into powder, and packaged into polythene bags. Moisture level in the dried leaves and pH were determined. Minerals in the leaf powder were determined by Atomic Absorption Spectrophotometry (AAS). Beta‐carotene levels were determined by spectrophotometry. Acceptability tests of dishes fortified with *M. oleifera* leaf powder were conducted with score sheets (Smileys) on a 5‐point hedonic scale of “Like Extremely” to “Dislike Extremely”, and dishes ranked to determine preference. Dried *M. oleifera* leaves contained high levels of micronutrients: 0.36 ± 0.04 mg/100 g Copper (Cu); 5.80 ± 0.68 mg/100 g Manganese (Mn); 20.96 ± 1.37 mg/100 g Iron (Fe); 6.79 ± 1.82 mg/100 g Zn; and 21.42 ± 1.67 mg/100 g *β*‐carotene. The dishes showed significant levels of these minerals compared to the nonfortified dishes (*P *< 0.05). *M. oleifera* leaf‐fortified dishes were also highly acceptable to the children (highest mean score of 5.0 ± 0 of 5 and lowest of 3.50 ± 1.43 of 5). *M. oleifera* leaf powder contains high levels of the micronutrients *β*‐carotene, Zn, Mn, and Fe, comparable to levels found in amaranth and spinach. *M. oleifera* leaf‐fortified local dishes were well accepted by children in Ada‐East district. *M. oleifera* leaf fortified dishes could be good sources of *β*‐carotene and other minerals for children vulnerable to malnutrition in Ghana.

## Introduction

Dark green leafy vegetables are rich sources of provitamin A carotenoids, vitamin C, folic acid, and dietary minerals such as iron, calcium, zinc, and manganese (Singh et al. 2001; Seshadri and Nambiar 2003; Sheela et al. 2004; Fahey 2005; Odhav et al. 2007; Raju et al. 2007; Oduro et al. 2008). These micronutrients are essential to the human body because they perform important biochemical roles needed for general well‐being. Iron (Fe) deficiency, for example, leads to iron deficiency disorders including anemia which affects about a third of the world's population (Senesse et al. 2004); vitamin A deficiency is associated with vitamin A deficiency disorders including night blindness, increased risk of resistance to severe infection and impaired embrayonic development and spermatogenesis in males (Clagett‐Dame and Knutson 2011; World Health Organization (WHO), 1995). Zinc deficiency contributes to the death of 800,000 children globally per year (Hagan et al. 2010). Deficiency of copper, zinc, and manganese impairs cell functions and affects growth and development, immune system, and metabolism. Copper is the primary constituent of cuproenzymes, which are involved in energy production, iron metabolism, and neurotransmitter synthesis metabolism (Mejía‐Rodríguez et al. 2013). Zinc is also important in neurologic function (Yamada et al. 2014). Globally, 195 million preschool children are vitamin A deficient (Aguayo and Baker 2005; World Health Organisation 2009). Adequate consumption of dark green leafy vegetables would supply *β*‐carotene and minerals that will contribute to prevention of disorders in children and adults.

In Ghana, green leafy vegetables such as African spinach, amaranth, leafy eggplant, *tossa,* and drumstick (*M. oleifera*) leaves are cultivated all year round, and thus are available for daily consumption. Ada‐East district is one of the rural districts in Ghana. Predominantly, the indigenous people are farmers and fishermen. Most of them cultivate onions, pepper, tomatoes, and cassava. (Ada East District Assembly 2013). A previous study (Glover‐Amengor and Vowotor 2013), however, indicated that *M. oleifera* is grown and sparingly consumed in the district, and could therefore, be promoted for food use. Dried drumstick leaves (*M. oleifera*) retained 87.5% and 50% of its *β*‐carotene after 4 and 3 month storage, respectively, and could therefore be processed for convenient use. (Glover‐Amengor et al. 2012; Seshadri et al. 1997). Fortifying the diets of rural households with *M. oleifera* leaves could serve as a less expensive way of improving their nutritional status, because the leaves could be cheaply dried with solar dryers and stored for household use. However, *M. oleifera* leaves are eaten in soups and stew mostly. Dietary innovations that incorporate *M. oleifera* leaves into other local dishes will increase and diversify the consumption of this green leafy vegetable. Consumer acceptability of the fortified dishes is key in the whole fortification process. Thus testing the consumer acceptability of dried *M. oleifera* leaves by incorporating it into local foods, and assessing the feasibility of introducing it into a school menu, will determine its potential for use as a food fortificant.

This study, which is part of an efficacy trial in which children were supplemented with dried *M. oleifera* leaves to determine its effect on vitamin A status, assessed.

1. The micronutrient composition *M. oleifera* leaf‐fortified dishes
2. Acceptability of *M. oleifera* leaf‐fortified dishes of children in Ada‐East district, Ghana.
3. Feasibility of introducing *M. oleifera* leaf‐fortified dishes into school menu of children in Ada‐East district, Ghana.

## Materials and Methods

### Leaf processing and nutrient determination

#### Materials

*M. oleifera* leaves, an ultraviolet (UV)‐proof solar dryer and stainless steel hammer mill.

*M. oleifera* leaves were harvested early in the morning (to ensure maximum nutrient conservation) from a field in Accra in the months of March and April, 2012. Leaves were sampled randomly from the field for three times at 2 weeks intervals (three replications). About 40 Kg of fresh leaves were collected per replication. The leaves were transported on ice to the processing hall of Council for Scientific and Industrial Research‐Food Research Institute (CSIR‐FRI).

Leaves were washed first in tap water, then in I% saline for three minutes to reduce the microbial load, and again in tap water (Ghana Standard Authority 2016). These leaves were then thinly spread on aluminum trays and dried between 35°C to 55°C in a UV‐proof solar dryer for 5 h (Glover‐Amengor and Mensah 2012). Duplicate samples of dried leaves were analyzed for various parameters. Moisture in the dried leaves was determined as described in AOAC 925.10 (AOAC 1990), pH was measured with a pH Meter (PHM 92. Radiometer Analytical A/S, Bagsvaerd, Denmark). A slurry was prepared from 10 g leaf powder and 10 mL distilled water. The pH meter was inserted into the slurry and the value read. Two readings were taken, and the average value was recorded. Measuring the pH was to ensure that the leaves did not deteriorate before drying, as this could affect protein and beta‐carotene levels, and also the taste of the products. Dried *M. oleifera* leaves contain about 20% protein, but this was not determined in this study which focused on micronutrients. Minerals were determined by Atomic Absorption Spectrophotometry (AAS) using dry ashing method (AOAC 2005). Beta‐carotene levels were measured spectrophotometrically (Karnjanawipagul et al. 2010). Replicate samples were analyzed separately for their micronutrient contents. The replicates were thoroughly mixed together and then milled using a locally fabricated stainless steel hammer mill (0.8 mm particle size). The milled product was packaged in clean polythene bags and kept at room temperature (28°C) in a wooden cupboard.

### Incorporation of *M. oleifera* leaf powder into various local dishes

Various local dishes were standardized and fortified with *M. oleifera* leaf powder at three levels (2 g, 3 g, and 5 g leaf powder/100 g product) in the test kitchen of CSIR‐Food Research Institute (CSIR‐FRI). The dishes included *ofam* (baked ripe plantain‐roasted maize meal blend), beans and *gari* (roasted cassava grits), *waakye (*boiled red cowpea and rice); groundnut soup, porridge (composite white maize, groundnut, and white cowpea meal), *nkontomire* (cocoyam leaves) sauce, *jollof* rice and *apapransa* (boiled cowpea and maize flour meal). Apart from porridge, *apapransa* and *ofam,* the other dishes formed part of the menu of schools participating in the Government of Ghana School Feeding Programme (GSFP) in Ada‐East district, Ghana. The leaf powder was weighed and added to an equivalent weight of the dish when almost done, and allowed to cook for five minutes. The heat treatment cooked the leaf powder and enhanced the bioavailability of *β*‐carotene. The dishes, eight in all, were then individually assessed in‐house for sensory attributes of taste, color, texture, appearance, flavor, aftertaste and overall acceptability on a 9‐point hedonic scale by ten semitrained, adult panelists. After in‐house acceptability testing, 2 g and 3 g levels were selected for child acceptability test as a multiple range analysis of the sensory attributes showed that increased concentration of *M. oleifera* leaf powder significantly affected the color of some of the dishes, and in some cases, appearance, taste, aftertaste, and flavor (Table 1). The dishes were up‐scaled to provide the needed quantity for acceptability testing. After child acceptability test, three dishes – porridge, *waakye,* and groundnut soup were selected for the efficacy trial. These dishes were highly preferred by the children (Table 4). These dishes were then fortified with *M. oleifera* leaf powder at 3 g/100 g and their micronutrient levels were assessed. Micronutrient levels of nonfortified dishes were also assessed.

**Table 1:** Multiple range analysis for attributes of *M. oleifera* leaf fortified dishes by adult judges

Food Sample	Appearance	Color	Texture	Flavor	Taste	Aftertaste	Overall acceptability
Porridge 3 g	4.00 ± 0.88^c^	7.10 ± 0.97^a^	6.50 ± 0.82^ab^	7.00 ± 0.95^a^	6.30 ± 1.17^a^	6.40 ± 1.07^ab^	6.40 ± 1.35^a^
Porridge 2 g	5.00 ± 1.06^b^	7.70 ± 1.03^a^	7.20 ± 1.15^a^	7.00 ± 1.45^a^	7.10 ± 1.69^a^	7.20 ± 1.25^a^	7.00 ± 1.35^a^
Porridge 5 g	6.00 ± 1.10^a^	5.90 ± 1.03^b^	5.80 ± 1.06^b^	6.70 ± 1.20^a^	6.10 ± 1.20^a^	5.90 ± 1.52^b^	5.90 ± 1.25^a^
*Ofam* 2 g	6.80 ± 1.23^a^	6.60 ± 1.26^a^	6.80 ± 1.32^a^	7.10 ± 1.10^a^	6.80 ± 1.62^a^	6.80 ± 1.32^a^	7.10 ± 1.52^a^
*Ofam* 3 g	6.60 ± 1.07^a^	6.30 ± 1.06^ab^	7.20 ± 1.03^a^	6.90 ± 1.37^a^	7.00 ± 1.15^a^	7.20 ± 1.03^a^	7.10 ± 1.10^a^
*Ofam* 5 g	5.90 ± 0.88^a^	5.30 ± 1.25^b^	6.20 ± 1.23^a^	6.00 ± 1.41^a^	6.40 ± 1.26^a^	6.30 ± 0.95^a^	6.20 ± 1.03^a^
Beans and *gari* 2 g	7.30 ± 0.95^a^	7.60 ± 0.70^a^	7.00 ± 0.82^a^	6.60 ± 1.35^a^	5.80 ± 1.40^a^	6.10 ± 1.52^a^	6.60 ± 1.43^a^
Beans and *gari* 3 g	7.20 ± 0.95^a^	7.30 ± 1.06^a^	6.70 ± 1.06^a^	6.20 ± 0.92^a^	5.10 ± 1.91^a^	4.60 ± 1.65^a^	5.10 ± 1.79^a^
Beans and *gari* 5 g	6.90 ± 1.37^a^	7.30 ± 1.34^a^	6.50 ± 1.08^a^	5.80 ± 1.48^a^	5.10 ± 1.97^a^	4.80 ± 1.99^a^	5.30 ± 1.95^a^
*Apapransa* 2 g	7.38 ± 1.00^a^	7.63 ± 0.88^a^	7.38 ± 0.88^a^	6.75 ± 1.39^a^	6.50 ± 1.42^a^	6.63 ± 1.69^a^	6.78 ± 1.48^a^
*Apapransa* 3 g	4.55 ± 2.13^b^	4.56 ± 2.19^b^	5.22 ± 1.48^b^	5.22 ± 2.11^a^	4.67 ± 1.41^b^	4.22 ± 1.48^b^	4.33 ± 1.58^b^
*Apapransa* 5 g	3.56 ± 1.67^b^	3.89 ± 1.90^b^	6.22 ± 1.39^ab^	5.33 ± 1.80^a^	4.33 ± 1.58^b^	4.33 ± 1.32^b^	4.22 ± 1.20^b^
*Nkontomire* sauce 2 g	7.60 ± 0.52^a^	7.50 ± 0.53^a^	6.90 ± 0.74^a^	6.90 ± 1.20^a^	6.90 ± 1.60^a^	6.80 ± 1.40^a^	7.20 ± 1.55^a^
*Nkontomire* sauce 3 g	6.70 ± 0.67^b^	6.80 ± 0.63^b^	6.50 ± 1.27^a^	5.90 ± 1.29^ab^	4.50 ± 1.43^b^	4.80 ± 1.14^b^	5.70 ± 1.18^b^
*Nkontomire* sauce 5 g	6.50 ± 0.71^b^	6.50 ± 0.71^b^	6.60 ± 0.84^a^	5.70 ± 1.25^b^	4.20 ± 0.79^b^	4.60 ± 1.58^b^	5.50 ± 1.06^b^
Groundnut soup 3 g	7.33 ± 0.87^a^	7.33 ± 1.00^a^	6.67 ± 1.00^a^	7.33 ± 1.22^a^	6.67 ± 1.41^a^	6.44 ± 1.13^a^	6.78 ± 0.97^ab^
Groundnut soup 2 g	7.22 ± 1.09^a^	7.56 ± 0.73^a^	7.00 ± 1.81^a^	7.11 ± 0.93^a^	6.67 ± 1.58^a^	6.22 ± 1.48^a^	7.99 ± 0.87^a^
Groundnut soup 5 g	6.11 ± 1.83^a^	6.00 ± 1.80^b^	6.44 ± 1.81^a^	6.00 ± 2.06^a^	5.56 ± 2.19^a^	5.67 ± 2.06^a^	5.44 ± 2.07^b^
*Waakye* 2 g	6.67 ± 1.50^a^	6.44 ± 1.42^a^	7.00 ± 1.41^a^	6.89 ± 1.05^a^	7.00 ± 1.12^a^	6.56 ± 1.42^a^	7.00 ± 1.73^a^
*Waakye* 3 g	4.89 ± 2.15^b^	4.56 ± 2.01^b^	6.11 ± 1.76^a^	5.33 ± 1.58^b^	6.00 ± 1.87^ab^	5.56 ± 2.01^ab^	5.89 ± 1.76^ab^
*Waakye* 5 g	4.56 ± 1.59^b^	3.89 ± 1.69^b^	6.11 ± 1.83^a^	5.11 ± 1.62^b^	4.56 ± 1.42^b^	4.44 ± 1.51^b^	4.56 ± 1.42^b^
*Jollof* rice 2 g	6.11 ± 1.62^a^	6.44 ± 1.33^a^	6.67 ± 1.12^a^	6.11 ± 1.54^a^	6.44 ± 1.51^a^	5.78 ± 1.99^a^	6.22 ± 1.64^a^
*Jollof* rice 5 g	3.78 ± 1.79^b^	3.78 ± 2.11^b^	5.44 ± 2.01^a^	5.33 ± 2.45^a^	5.56 ± 1.67^ab^	5.00 ± 2.06^a^	5.78 ± 1.92^ab^
*Jollof* rice 3 g	2.67 ± 2.06^b^	2.78 ± 2.05^b^	6.33 ± 1.80^a^	4.44 ± 2.65^a^	3.89 ± 2.09^b^	3.00 ± 2.00^b^	3.78 ± 2.64^b^

ANOVA was used to determine if a difference existed among the sample attributes and Fisher's test (least significance test) was used to determine how different the various samples were from each other.

Means with the same superscript are not significantly different (*P* < 0.05).

### Acceptability test by school children

#### Sampling method/procedure

A sample size of thirty‐eight (38) was used (Nambiar et al. 2003). The school for the study was purposively selected in a village called Luhuese because it had a kindergarten and was also participating in GSFP. Class registers were pooled together from kindergarten to class three to obtain a sampling frame. All eligible children (ages 4–12 years), present in school on the day of testing and whose caregivers had given ascent to their participation (*N* = 101) were listed. Since the sample size was thirty‐eight (38), 38 “Yes” and 63 “No” were written on pieces of paper, folded well and mixed thoroughly in a box. All children who picked “Yes” were admitted into the study, whereas “No” was rejected.

The various dishes were coded with three digit random numbers generated with Excel. The children's names were written, but each was given alphabetical codes only known to the research team. Given that each child will taste three dishes only, the number of children that tasted each dish was generated with Excel Stat.

The children assessed eight (8) dishes (but nine samples) comprising both sweet (porridge and *ofam*) and salty dishes: *waakye*,* jollof* rice, *apapransa*, beans with *gari*, groundnut soup, and *nkontomire* sauce. *Nkontomire* sauce was fortified at 2 g/100 g and also 3 g/100 g General acceptability was evaluated using score sheets with facial expressions (smiley’s) depicting a 5‐point hedonic scale of “Like Extremely” through “Dislike Extremely”. It was a school‐based study, so the children, assisted by the research team, assessed the dishes in their classrooms at the village called Luhuese. One sample was served at a time, and about 2 g food was given to a child. Hot food was transported to the school in food warmers, so the samples were hot at the time of serving. Ethical clearance for the study was obtained from the Institutional Review Board (IRB) of the Noguchi Memorial Institute for Medical Research (NMIMR). Parents willing to allow their children to participate in the study either signed or thumb‐printed an ascent form after the objectives and benefits of the study were explained to them.

After the acceptability study, the children were further supplemented for 2 weeks with *M. oleifera* leaf‐fortified *waakye,* groundnut *soup or jollof* rice to assess the feasibility of introducing dried leaves into the school menu. Unlike the acceptability study which was cross‐sectional, the feasibility study was interventional, in that it was not conducted once, but children were supplemented, three times a week, for 2 weeks. Each child received 2 g leaves three times a week. The meals formed part of the regular menu served the children in this school which was one of the schools participating in the GSFP. The leaves were incorporated into about 150 g food, the normal portion served each child. The supplementation trial was assessed by consumption of the portions allocated to the children, left‐overs (plate waste, no plate waste) and presence in school during the intervention period.

### Statistical analysis

Statistical analysis was done using Statistical Package for Social Scientists (SPSS 16 for Windows, 2005) version 16.0. Analysis of Variance (ANOVA) and a multiple range test (Duncan test) were conducted at a level of significance of *P* < 0.05 to test for significant differences between means of nutrients in fortified and nonfortified dishes. For the child acceptability test, a generalized linear model (GLM) was used for the calculations. Analysis of Variance (ANOVA) was used to determine if a difference existed among the sample scores, and Fisher's test (least significance test) was used to determine how different the various samples were from each other.

## Results

A multiple range analysis for attributes of *M. oleifera* leaf fortified dishes by an in‐house semitrained adult judges is shown in Table 1. Increasing concentrations of *M. oleifera* leaf powder significantly affected the appearance, taste, aftertaste, and overall acceptability of some of the dishes. There were no differences in overall acceptability of porridge with increasing concentration of *M. oleifera* leaf powder. Concentration did not have any significant effect on color at 2 g and 3 g levels, but a significant difference existed at 5 g fortification level. With *Nkontomire* sauce which is another green leafy vegetable, although there was a significant difference between the color at 2 g and 3 g levels, there was no significant difference between 3 g and 5 g levels.

The *β* ‐carotene, copper, zinc, iron, and manganese levels in dried *M. oleifera* leaves are shown in Table 2. The Table shows that *M. oleifera* leaves contained various levels of the micronutrients *β*‐carotene, Cu, Zn, Mn, and Fe. Table 3 shows levels of the micronutrients in the fortified and nonfortified dishes. Fortification significantly increased levels of all the micronutrients except Zn (*P* < 0.05). Fortified porridge, for example, contained 1.28 mg/100 g *β*‐carotene as against 0.02 mg/100 g in the nonfortified dish. Similarly, fortified *waakye* contained 2.35 mg/100 g iron as against 0.96 mg/100 g in the nonfortified dish. For *waakye*, the fortification resulted in a significant increase in all the micronutrients. While fortification of soup led to a significant increase in levels of Fe and Mn, there was no change in Zn levels. The result of the acceptability test of *M. oleifera* leaf‐fortified dishes is shown in Table 4. The children's acceptability test rated porridge as the most preferred dish among all the others. Moreover, all the other dishes were also highly acceptable to the children. Porridge, beans and *gari,* groundnut soup, *waakye*,* nkontomire* sauce (2 g/100 g) had mean scores ranging from 4.64 to 5 implying that they were liked extremely, whereas all the remaining dishes had mean values indicating that they were also liked very much. In the feasibility study, the children ate all their portions during the 2‐week intervention period, and there were no plate wastes either. Compliance was 100 percent.

**Table 2:** Mineral and *β*‐carotene content of dried *M. oleifera* leaves

Replicate	Sample	Cu (mg/100 g)	Fe (mg/100 g)	Mn (mg/100 g)	Zn (mg/100 g)	*β*‐carotene (mg/100 g)	Moisture (%)	pH
1	*Moringa leaves*	0.40 ± 0.18^a^	19.49 ± 2.99^a^	5.20 ± 0.21^a^	5.38 ± 0.25^a^	20.79 ± 0.01^a^	7.61 ± 0.07^a^	5.34 ± 0.01^b^
2	*Moringa leaves*	0.37 ± 0.09^a^	22.20 ± 11.91^b^	5.67 ± 4.56^ab^	6.14 ± 0.23^b^	20.15 ± 0.00^b^	7.58 ± 0.02^a^	5.32 ± 0.04^b^
3	*Moringa leaves*	0.32 ± 0.04^b^	21.20 ± 2.43^bc^	6.54 ± 1.84^b^	8.85 ± 0.23^c^	23.31 ± 0.01^c^	7.73 ± 0.04^a^	5.35 ± 0.01^b^
	*Mean of Reps*	0.36 ± 0.04	20.96 ± 1.37	5.80 ± 0.68	6.79 ± 1.82	21.42 ± 1.67	7.64 ± 0.08	5.34 ± 0.02

1, 2, 3 are replications of sample. Means with the same superscript are not significantly different (*P* < 0.05).

Cu, Copper; Fe, Iron; Mn, Manganese; Zn, Zinc.

**Table 3:** Mineral and *β*‐carotene content of dishes

Sample	Food	Cu (mg/100 g)	Fe (mg/100 g)	Mn (mg/100 g)	Zn (mg/100 g)	*β*‐carotene (mg/100 g)
1	Porridge	0.09 ± 0.11^a^	0.36 ± 0.49^d^	0.15 ± 0.17^b^	0.40 ± 0.46^f^	0.02 ± 0.00^a^
2	Porridge	0.15 ± 0.13^c^	2.13 ± 1.64^g^	0.26 ± 0.08^d^	0.39 ± 0.21^f^	1.28 ± 0.04^c^
1	*Waakye*	0.10 ± 0.03^ab^	0.96 ± 1.28^e^	0.10 ± 0.01^a^	0.56 ± 0.62^g^	0.08 ± 0.05^a^
2	*Waakye*	0.14 ± 0.22^bc^	2.35 ± 3.83^g^	0.62 ± 0.31^e^	0.78 ± 0.69^h^	0.94 ± 0.51^bc^
1	Soup	0.10 ± 0.13^ab^	1.54 ± 0.69^f^	0.20 ± 0.04^c^	0.40 ± 0.36^f^	0.34 ± 0.06^ab^
2	Soup	0.10 ± 0.26^ab^	2.62 ± 2.43^g^	0.26 ± 0.30^d^	0.43 ± 0.16^f^	2.98 ± 0.39^d^

1 = Nonfortified; 2 = M. oleifera leaf fortified. Dishes were fortified at 3 g/100 g product. Means with the same superscript are not significantly different (*P* < 0.05).

Cu, Copper; Fe, Iron; Mn, Manganese; Zn, Zinc.

**Table 4:** Acceptability of *M. oleifera* leaf‐fortified dishes

Dish	Mean + S.D	N
Porridge (2 g/100 g)	5.0 ± 0^a^	13
*Waakye* (2 g/100 g)	4.88 ± 0.33^a^	12
*Nkontomire* sauce (2 g/100 g)	4.80 ± 0.42^ab^	12
Groundnut soup (2 g/100 g)	4.71 ± 0.83^ab^	13
Beans and *gari* (2 g/100 g)	4.64 ± 0.50^ab^	13
*Apranpransa* (2 g/100 g)	4.40 ± 0.97^ab^	13
*Jollof* rice(2 g/100 g)	4.36 ± 0.92^ab^	12
*Ofam* (2 g/100 g)	4.18 ± 1.33^b^	13
*Nkontomire* sauce (3 g/100 g)	4.50 ± 1.43^c^	13

ANOVA was used to determine if a difference existed among the sample scores, and Fisher's test (least significance test) was used to determine how different the various samples were from each other. *P* = 0.0026. Thus *P *< 0.05 means with the same superscript are not significantly different (*P* < 0.05).

## Discussion

The *M. oleifera* leaves sampled contained all the micronutrients tested (*β* –carotene, Cu, Zn, Mn, and Fe) in amounts comparable to the findings of other workers (Seshadri and Nambiar 2003; Glover‐Amengor and Mensah 2012; Moyo et al. 2011; Ogbe and Affiku 2011; Fuglie 1999). These levels were reflected in the fortified dishes which showed significantly higher levels of micronutrients than the nonfortified dishes (Table 3). Leaf‐fortified porridge (Porridge 2), for example, contained 0.15 mg/100 g Cu, 0.26 mg/100 g Mn, and 2.13 mg/100 g Fe. The nonfortified dishes, however, contained 0.09 mg/100 g Cu, 0.15 mg/100 g Mn, and 0.36 mg/100 g Fe, respectively. The implication of these findings is that *M. oleifera* leaves could be used to increase the levels of Cu, Fe, Mn, Zn, and *β*–carotene of diets.

Porridge can be instantly prepared with cereal/legume composite flour and fed to children, hence its preference over other dishes further enhances its use as a vehicle for *M. oleifera* leaf delivery. Interestingly, when supplemented for 2 weeks, the children still found the fortified dishes acceptable, and ate their portions over the supplementation period. Nambiar et al. (2003) also assessed the feasibility and acceptability of introducing dehydrated *M. oleifera* leaves as a source of vitamin A among 40 preschool children in an integrated child development program. They also found that the fortified dishes (5–7 g/100 g product) were highly acceptable to the children.

The implication of these results for food‐to food fortification is that *M. oleifera* leaves have the potential to serve as a less expensive *β*‐carotene and mineral source in the diets of children in Ghana and other tropical countries where these vegetables grow and adapt easily and where children often have marginal vitamin A status (Singh et al. 2001; Manh et al. 2005) and also suffer from iron deficiency anemia in addition to other mineral deficiencies.

The study showed other *M. oleifera* leaf‐fortified dishes such as *waakye,* groundnut soup and *jollof* rice were also highly acceptable to the children during the 2‐week fortification of their school GSFP menu, and they (100%) ate their portions of food during the period. This is significant because the plant could be grown in backyards for household use. Also, the GSFP could include *M. oleifera* leaves for enriching the diets of the children.

Although *β*‐carotene is fat soluble it does not pose any toxicity challenges, because *β*‐carotene is stored safely and only converted to vitamin A when the body's vitamin A stores are depleted (Wardlaw 1999). *M. oleifera* leaf consumption would thus ensure adequate reserves of *β*‐carotene that could become available for conversion when needed by the body. 10 g of dried *M. oleifera* leaves a day could provide 50–100% of the vitamin A needs of all categories of age brackets and about 30% of the iron needs of children between 1 and 12 years (Saveur et al. 2010; Food and Nutrition Board, Institute of Medicine, National Academies, 2005). All the other nutrients including protein would also become available to the child (Kouevi 2013).

## Conclusion

In conclusion, the study has shown that, *M. oleifera* leaf powder contains significant levels of the micronutrients *β*‐carotene, Cu, Zn, Mn, and Fe. Supplementation of local dishes resulted in significant levels of the micronutrients in the fortified dishes. The study further showed that school children found all the fortified dishes highly acceptable. Thus when promoted and adopted, these *M. oleifera* leaf fortified dishes could be good sources of *β*‐carotene and other minerals for tropical countries.

## Conflict of Interest

None declared.
